# Comparison of Price Index Methods and Drug Price Inflation Estimates for Hepatitis C Virus Medications

**DOI:** 10.1001/jamahealthforum.2023.1317

**Published:** 2023-06-09

**Authors:** T. Joseph Mattingly, Gerard F. Anderson, Joseph F. Levy

**Affiliations:** 1 Department of Pharmacotherapy, University of Utah College of Pharmacy, Salt Lake City; 2 Department of Health Policy and Management, Johns Hopkins Bloomberg School of Public Health, Baltimore, Maryland

## Abstract

**Importance:**

Measuring drug price inflation is challenging because new drugs continually enter the market, some drugs transition from branded to generic, and current inflation indexes do not account for these market basket changes. Instead, they measure the price increases after new drugs have been launched. Therefore, the public pays the higher costs of newer and usually more expensive drugs, but the inflation indexes do not reflect the increases over existing drugs previously used to treat the same conditions.

**Objective:**

To assess how price index methods can affect estimates of drug price inflation using a case study of hepatitis C virus (HCV) medication and to explore other approaches for constructing a price index.

**Design, Setting, and Participants:**

This cross-sectional study used data from outpatient pharmacies to compile a list of all HCV medications that were ever on the market (brand and generic) from 2013 to 2020. Using National Drug Codes of HCV drugs, a 20% nationally representative sample of Medicare Part D claims from 2013 to 2020 was queried. Alternative drug price indexes, including product-level vs class-level product and quantity definitions were developed in which gross vs net price definitions were used and an adjustment was created and applied to capture treatment duration because newer drugs often required a shorter duration.

**Main Outcomes and Measures:**

Price index value and rate of inflation from 2013 to 2020 for each methodologic approach to constructing a drug pricing index.

**Results:**

In all, 27 different HCV drug regimens were identified in Medicare Part D claims in 2013 to 2020. A product-level approach for measuring inflation estimated a 10% gross drug price increase from 2013 to 2020 for HCV drugs, whereas a class-level approach including the higher prices of the new drugs showed a 31% gross price increase. After adjusting for manufacturer rebates to estimate net prices, the findings showed that HCV drug prices fell by 31% from 2013 to 2020.

**Conclusions and Relevance:**

The findings of this cross-sectional study indicate that the current product-level methods to estimate drug price inflation underestimated price increases for HCV drugs by failing to include the high launch prices of new market entrants. Using a class-level approach, the index captured higher spending on new products at launch. Prescription-level analyses, which did not consider shorter durations of treatment, overestimated price increases.

## Key Points

**Question:**

Which methodologic considerations affect drug price inflation estimates, and what may be a better approach to constructing a drug price index?

**Findings:**

In this cross-sectional study of drug price indexes, including a case study of hepatitis C virus medications, a product-level index failed to capture high-cost drugs at launch compared with a class-level index approach. Additionally, adjusting for prescription duration and manufacturer rebates substantially affected inflation rate calculations.

**Meaning:**

The findings of this cross-sectional study indicate that although the Inflation Reduction Act prevents prescription drug prices from outpacing the Consumer Price Index, it fails to address new drug launches for specific diseases, which can substantially increase the average cost of treatment for patients.

## Teaser

This cross-sectional study identifies the shortcomings of current drug pricing indexes to estimate drug price inflation and considers a new approach.

## Introduction

Drug price inflation has recently received substantial attention from patients, clinicians, researchers, and policy makers who are concerned with affordability of medications. The challenge in measuring the rate of inflation for drugs is that new drugs continually enter the market and drugs transition from branded to generic, yet the current inflation indexes do not account for these market basket changes. Current inflation indexes do not capture a new drug until it has at least 2 periods of data; therefore, the patient experiences the typically higher cost of a newer drug to treat the same condition, while the inflation indexes lag behind, not capturing the increase until they have additional data.

The 117th US Congress explicitly targeted prescription drug price inflation with the passage of the Inflation Reduction Act of 2022 (IRA). One of the drug pricing provisions in the IRA states that the Average Sales Price (ASP) or the Average Manufacturer’s Price (AMP) of any drugs sold through Medicare Parts B and D cannot increase more than overall inflation.^1^ The Centers for Medicare & Medicaid Services will calculate the Part D inflation rebate amount separately for each dosage form and strength using quarter-weighted AMP data to calculate an Annual Manufacturer Price—the average price at which manufacturers sold the product during the applicable 12-month period.^2^ The inflation metric selected for comparison is the Consumer Price Index for All Urban Consumers—a measure of average changes in prices over time for urban consumers for a fixed market basket of consumer goods and services.^3^

Although this mechanism keeps drug prices below the rate of inflation, the public may still experience increases in drug costs that are greater than overall inflation. When new drugs enter the market at prices higher than the drugs they replace, these higher launch prices are not included in the current inflation indexes. The new drug price will not be reflected in the inflation index until after the launch and only then will the indexes measure price increases (or decreases). That is, patients will pay a much higher price for a new drug, but the inflation index will not measure the increased cost to the patient; instead, it will measure any postlaunch fluctuations in price.

Researchers and the government have developed multifarious ways to measure inflation broadly, in addition to the CPI approach.^4,5^ Price indexes have been developed using a variety of weighting methods (eg, Laspeyres, Paasche, Fisher) and different definitions of *price* and *quantity* for the goods and services selected in creating the market basket. Calculating the rate of inflation for drugs is more methodologically difficult than calculating the rate of increase for most other medical services or goods and services overall. The US Bureau of Labor Statistics calculates an inflation rate for prescription and nonprescription drugs,^6^ using a fixed sample of drugs. Numerous concerns have been expressed regarding the validity of this approach, including the small number of drugs in the sample and the point that the indexes do not reflect what patients pay. Bosworth and colleagues proposed an alternative method using actual dispensing data, an approach that demonstrated more rapid rates of price increases than shown by the Bureau of Labor Statistics approach.^7^

There are 3 main challenges to creating an inflation measure for drugs. First and most important is the continuously changing number and types of drugs available. This is a dynamic market where new drugs enter, others are withdrawn, and some formulations transition from being exclusively branded to having generic alternatives. Most health care inflation indexes use a fixed market basket of goods and services that does not change appreciably over time. For example, hospitals use approximately the same percentage of nursing staff, laboratory tests, and capital during a 10-year period. As new drugs enter the market with substantially higher prices than existing drugs, overall drug expenditures increase. However, unless the market basket measures the higher prices of the new drugs, the inflation measure will underestimate the cost to the patient. When new drugs are incorporated into the market basket after launch, price increases are only measured postlaunch and the higher price of the new drug is not reflected by the inflation index. If the manufacturer of the new drug lowers its price postlaunch, the inflation index will go down even when patients and insurers are paying more for a new drug to treat the same condition.

Second, drugs have a myriad of prices. An inflation index may be making comparisons among the manufacturer’s list price, wholesale acquisition cost, the National Average Drug Acquisition Cost, the average wholesale price, manufacturer net price after rebates, ASP, and/or the usual and customary price.^8^ All of these prices have different meanings and potentially different spending and price trajectories. Each price measures a different phase in the supply chain.

Third, although an index can be constructed to adjust for improvements in convenience or quality of goods or services—as hedonic price indexes do—the most common inflation indexes do not make these adjustments.^9,10^ For pharmaceutical medications, this would measure the treatment duration required for each new drug and its effectiveness compared with the existing drug. Because drugs vary in effectiveness, comparing drugs from 2000 with drugs in 2022 presents challenges. In addition, most measures of drug price inflation tend to incorrectly assume that the duration of treatment and the quality of the product or service has not changed over time.

The aim of this study was to understand how these 3 challenges typically affect the rate of drug price inflation and to apply 5 methodologic considerations that are key to constructing a price index. We performed a case study using hepatitis C virus (HCV) treatment because it had undergone major therapeutic breakthroughs from 2013 to 2015, and its new HCV drugs entered the market at prices much higher than those previous drugs. We also aimed to provide recommendations for developing class-based indexes that reflect the actual price increases experienced by patients who are prescribed new drugs.

## Methods

This cross-sectional study followed the best practices recommended by the Strengthening the Reporting of Observational Studies in Epidemiology (STROBE) reporting guideline. The study was exempted from review because it did not use human participants and data were deidentified; informed consent was waived for the same reasons.

This study considered the various options for measuring drug inflation and 5 methodologic considerations important to constructing a price index: (1) product definition and substitution effects, (2) price definition, (3) quantity definition, (4) calculation approach, and (5) adjustment for quality improvements (Table 1).^11,12,13,14^ We applied these 5 considerations to evaluate how they affected the rate of increases observed for HCV drug prices in Medicare Part D from 2013 to 2020. We also created multiple drug price indexes, including product- vs class-level product and quantity definitions, gross vs net price definitions, and an adjustment to capture data on the duration of each HCV drug regimen, thereby accounting for the better quality and shorter treatment requirements of newer drugs.

**Table 1.** The 5 Key Methodologic Considerations for the Construction of a Drug Price Index

Index factor	Consideration and source
(1) Product definition	For consumer goods, this is straightforward; for example, purchasing a television. A tablet and an injection of the same drug product could be considered as a single product or as 2 different products. Another example is whether the branded and generic versions of a drug should be considered to be the same if they have the same therapeutic value. Some drug price indexes have been built at the molecule level to account for generic substitution. A third example is the drugs that treat patients with the same disease and are considered to be therapeutic substitutes.^11,12^
(2) Price definition	For most consumer goods, such as a television, retail prices are collected for comparison. There are many different prices in the pharmaceutical industry and each measures a different place in the drug supply chain (eg, list price, net price, patient out-of-pocket cost) and the price definition should align with the objective of the index.^9,13^
(3) Quantity definition	The most common approach, built on convenience, converts all products to quantities in 30-d prescriptions. Although this approach may be appropriate for long-term medications that are expected to be filled 12 times/year. For medications prescribed for acute conditions for a limited duration, this prescription-based approach will overestimate the price that a patient would expect to pay in each period, and it gives more weight to medications where more prescriptions are counted in the period.^6,14^
(4) Calculation approach	The Laspeyres, Paasche, and Fisher Price index methods have all been used in drug price index development. The method selected may over- or underestimate inflation based the product definition selected and how the market basket changes over time.^6,11,12^
(5) Adjustment for quality improvements	All price indexes are designed to measure price changes while holding quality constant. For many goods and services, inflation calculations based solely on price changes do not account for technological advances.^11^

### Study Data and Population

A list of all hepatitis C medications that were ever on the market from 2013 to 2020, including brand and generic drugs, was compiled. This list was based on HCV clinical guidelines (eTable 1 in Supplement 1), clinical practices procedures, and pharmacy expertise.^15,16,17,18,19^ A list of National Drug Codes (NDCs) for each drug used in the treatment was created using Red Book.^20^ From these NDCs codes, we queried a 20% nationally representative sample of Medicare Part D claims filed in 2013 to 2020. All data sets were deidentified before analyses. For each relevant claim, drug quantity and payment amount were recorded.

### Measures and Outcomes

The price measure was Medicare prerebate spending for each product identified in the Medicare Part D claims database as the plan paid amount. Different drugs have different treatment duration requirements that are based on clinical guidelines (eTable 1 in Supplement 1). For each product, we determined an appropriate annualized utilization quantity to reflect the 12-month duration needed for some drugs vs 2-month duration for others. By determining an *annualized prescription number* for our quantity definition, newer products requiring only 2 to 3 months of therapy would be appropriately compared with older 12-month therapies. Medicare receives confidential drug rebates. We used the gross-to-net discount rate derived from the SSR Health pricing tool^21^ to estimate postrebate net prices.

### Analyses

First, the standard *product-level* approach was used. With this approach, new drugs were not included because the market basket is fixed in the first year (annually). The prices and quantities at the beginning of the study period (2013) were fixed, and the price changes were measured in 2014. A common modification to this approach is called a chained index, in which new drugs are incorporated as they enter the market. However, when a new drug enters the market, there is no change in the index for that period. The reason for this is that both a simple Laspeyres and chained-Laspeyres method use the quantity of a product from the prior period to weight the price in the current quarter. The first (potentially very high) launch price does not enter the index until the second period.

Second, to be able to include the launch prices of new drugs in the index, a *class-level* approach using a chained-Laspeyres method was used. This approach combines all HCV products into a single class, and it is critical to capture the prices of the new product launches as well as all generic products available for consumers throughout the period. This class-based imputation method recognizes within-class substitution.

Third, to address changes in the duration of treatment, we calculated the indexes using both the actual prescriptions observed in the data along with an annualized prescription adjustment based on recommended durations per the clinical guidelines (eTable 1 in Supplement 1).

## Results

In this case study of HCV treatment, the Medicare Part D claims data (eTable 2 in Supplement 1) showed that several products were introduced and/or withdrawn from the market in 2013 to 2020. A summary of the medications included in the study sample and their average days’ supply, quantity dispensed, Medicare Part D paid amount, number of prescriptions dispensed, and number of adjusted annualized prescriptions dispensed are presented in eTables 2 and 3 in Supplement 1. Additional information is also available from the eReferences in Supplement 1.

**Figure 1.**
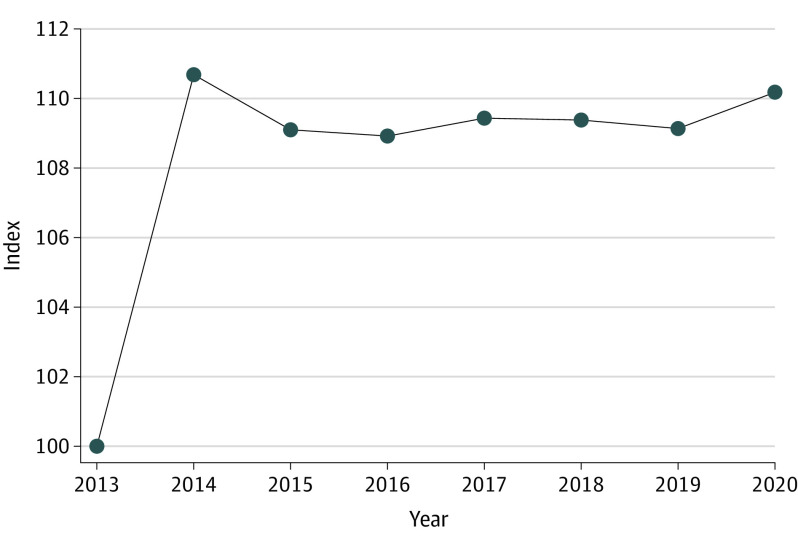
Conventionally Used Product-Level Gross Spending Index for Hepatitis C Virus (HCV) Drugs, 2013 to 2020

Using 2013 as the base year and the standard product-level approach, gross spending chained-Laspeyres price index showed that HCV drug prices were 10% greater in 2020 (Figure 1). An example of a limitation of the product-level index was that using 2013 as the base year failed to capture the influence of the $142 million (14.8% market share) spent on Harvoni (ledipasvir/sofosbuvir) in 2014 (Table 2).

**Table 2.** Product-Level Spending in 20% Medicare Part D Sample for Hepatitis C Virus Drugs, 2013 to 2020

Active ingredient (brand/generic)	2013 $ (%)	2014 $ (%)	2015 $ (%)	2016 $ (%)	2017 $ (%)	2018 $ (%)	2019 $ (%)	2020 $ (%)
Total spending (100%)	78 526 256	957 706 442	1 789 918 193	1 312 359 832	923 956 400	680 708 414	496 330 374	328 597 603
Ribavirin (Copegus)	57 371 (<1.0)	9149 (<1.0)	472 (<1.0)	NA	NA	NA	NA	NA
Daclatasvir (Daklinza)	NA	NA	25 620 338 (1.4)	82 951 825 (6.3)	27 387 991 (3.0)	1 090 417 (<1.0)	130 519 (<1.0)	NA
Sofosbuvir/Velpatasvir (Epclusa)	NA	NA	NA	77 772 808 (5.9)	188 363 792 (20.4)	182 993 648 (26.9)	198 963 472 (40.1)	177 384 848 (54.0)
Ledipasvir/Sofosbuvir (Harvoni)	NA	142 004 032 (14.8)	1 415 904 384 (79.1)	890 770 496 (67.9)	519 126 688 (56.2)	350 771 392 (51.5)	147 572 368 (29.7)	49 034 250 (14.9)
Telaprevir (Incivek)	35 298 472 (45.0)	1 605 699 (<1.0)	NA	NA	NA	NA	NA	NA
Ledipasvir/sofosbuvir (generic)	NA	NA	NA	NA	NA	NA	4 997 504 (1.0)	3 967 919 (1.2)
Glecaprevir/pibrentasvir (Mavyret)	NA	NA	NA	NA	14 548 845 (1.6)	94 495 840 (13.9)	110 620 192 (22.3)	58 644 657 (17.9)
Ribavirin (Moderiba)	NA	22 439 (<1.0)	8572 (<1.0)	5055 (<1.0)	NA	NA	NA	NA
Ribavirin (Moderiba 1200 dose pack)	NA	151 274 (<1.0)	74 091 (<1.0)	20 787 (<1.0)	NA	569 (<1.0)	NA	NA
Ribavirin (Moderiba 800 dose pack)	NA	19 498 (<1.0)	14 238 (<1.0)	3298 (<1.0)	NA	NA	NA	NA
Simeprevir (Olysio)	272 006 (<1.0)	167 268 400 (17.5)	23 285 510 (1.3)	5 021 470 (<1.0)	1 484 885 (<1.0)	43 081 (<1.0)	NA	NA
Peginterferon alfa-2a (Pegasys)	12 653 730 (16.1)	6 957 538 (<1.0)	1 448 472 (<1.0)	1 625 287 (<1.0)	2 074 094 (<1.0)	2 293 887 (<1.0)	2 884 711 (<1.0)	3 750 751 (1.1)
Peginterferon alfa-2a (Pegasys Proclick)	10 903 630 (13.9)	7 505 881 (<1.0)	248 925 (<1.0)	72 168 (<1.0)	130 335 (<1.0)	183 753 (<1.0)	105 680 (<1.0)	NA
Peginterferon alfa-2b (Pegintron)	4 131 999 (5.3)	4 520 409 (<1.0)	373 037 (<1.0)	30 551 (<1.0)	47 724 (<1.0)	36 503 (<1.0)	27 295 (<1.0)	NA
Ribavirin (Rebetol)	1073 (<1.0)	1591 (<1.0)	10 607 (<1.0)	59 (<1.0)	1386 (<1.0)	2870 (<1.0)	618 (<1.0)	NA
Ribavirin (Ribasphere)	557 101 (<1.0)	623 382 (<1.0)	468 735 (<1.0)	310 724 (<1.0)	93 230 (<1.0)	25 344 (<1.0)	355 (<1.0)	NA
Ribavirin (Ribaspher Ribapak)	1 550 185 (2.0)	3 323 044 (<1.0)	1 131 918 (<1.0)	492 172 (<1.0)	72 865 (<1.0)	5282 (<1.0)	NA	NA
Ribavirin (generic)	910 667 (1.2)	1 565 841 (<1.0)	1 393 859 (<1.0)	829 671 (<1.0)	366 469 (<1.0)	129 615 (<1.0)	78 459 (<1.0)	65 534 (<1.0)
Sofosbuvir/velpatasvir (generic)	NA	NA	NA	NA	NA	NA	10 400 508 (2.1)	21 691 460 (6.6)
Sofosbuvir (Sovaldi)	2 824 399 (3.6)	620 411 968 (64.8)	272 735 168 (15.2)	193 855 776 (14.8)	43 050 384 (4.7)	1 968 301 (<1.0)	662 828 (<1.0)	928 586 (<1.0)
Peginterferon alfa-2b (Sylatron)	600 843 (<1.0)	537 634 (<1.0)	772 169 (<1.0)	515 572 (<1.0)	221 134 (<1.0)	258 028 (<1.0)	121 247 (<1.0)	9513 (<1.0)
Ombitasvir/paritaprevir/ritonavir (Technivie)	NA	NA	648 431 (<1.0)	231 653 (<1.0)	55 006 (<1.0)	NA	NA	NA
Boceprevir (Victrelis)	8 764 780 (11.2)	1 150 333 (<1.0)	20 028 (<1.0)	NA	NA	NA	NA	NA
Dasabuvir/ombitasvir/paritaprevir/ritonavir (Viekira Pak)	NA	28 330 (<1.0)	45 759 240 (2.6)	16 206 543 (1.2)	1 926 229 (<1.0)	83 716 (<1.0)	NA	NA
Dasabuvir/ombitasvir/paritaprevir/ritonavir (Viekira XR)	NA	NA	NA	2 075 681 (<1.0)	4 148 325 (<1.0)	173 944 (<1.0)	NA	NA
Sofosbuvir/velpatasvir/voxilaprevir (Vosevi)	NA	NA	NA		16 467 711 (1.8)	30 031 932 (4.4)	18 532 040 (3.7)	12 869 031 (3.9)
Elbasvir/grazoprevir (Zepatier)	NA	NA	NA	39 568 237 (3.0)	104 389 310 (11.3)	16 120 293 (2.4)	1 232 579 (<1.0)	251 055 (<1.0)

Abbreviation: NA, not applicable.

Using a class-level approach, the chained-Laspeyres Price Index estimates HCV drug prices were nearly 500% greater in 2020 compared with the 2013 base year (Table 3), reflecting the higher prices of the new drugs. However, this is before accounting for the changes in prescription duration. After we incorporated adjustments for duration, the class-level, gross spending price index estimated that HCV drug prices were 31% greater in 2020 compared with the 2013 base year (Table 3).

**Table 3.** Class-Level Price Index, Unadjusted and Adjusted for Dosing Regimen for All Hepatitis C Virus Drugs, in Millions of US dollars ($M), 2013 to 2020

Spending	2013, $M	2014, $M	2015, $M	2016, $M	2017, $M	2018, $M	2019, $M	2020, $M
Total annual spending in Medicare Part D	78.5	957.7	1789.9	1312.4	924.0	680.7	496.3	328.6
Gross-to-net discount rate for HCV products	0.4095	0.1303	0.3870	0.5198	0.5225	0.6343	0.6518	0.6910
Net spending after rebate/discount adjustment	46.4	833.0	1097.2	630.3	441.2	249.0	172.8	101.5
**Quantity, y**	**2013**	**2014**	**2015**	**2016**	**2017**	**2018**	**2019**	**2020**
Total Rxs	21 334	54 557	70 677	52 744	37 830	29 474	24 910	17 900
Total annualized Rxs	2076	13 716	28 249	20 589	14 778	12 422	10 134	6629
**Price, y**	**2013**	**2014**	**2015**	**2016**	**2017**	**2018**	**2019**	**2020**
Gross spending/Rx	3681	17 554	25 325	24 882	24 424	23 095	19 925	18 357
Net spending per Rx	2174	15 268	15 524	11 949	11 662	8447	6939	5672
Gross spending per annualized Rx	37 818	69 826	63 361	63 740	62 525	54 801	48 978	49 570
Net spending per annualized Rx	22 332	60 731	38 841	30 611	29 855	20 043	17 056	15 317
**Indexes using actual Rx counts**								
Laspeyres Price Index (single year)	100	447	144	98	98	95	86	92
Gross Spending Index (2013-2020)	498.73	NA	NA	NA	NA	NA	NA	NA
Laspeyres Price Index (single year)	100	702	102	77	98	72	82	82
Net Spending Index (2013-2020)	260.98	NA	NA	NA	NA	NA	NA	NA
**Indexes using annualized Rx counts**								
Laspeyres Price Index (single year)	100	185	91	101	98	88	89	101
Gross Spending Index (2013-2020)	131.07	NA	NA	NA	NA	NA	NA	NA
Laspeyres Price Index (single year)	100	272	64	79	98	67	85	90
Net Spending Index (2013-2020)	68.59	NA	NA	NA	NA	NA	NA	NA

Abbreviations: HCV, hepatitis C virus; NA, not applicable; Rx, prescription.

**Figure 2.**
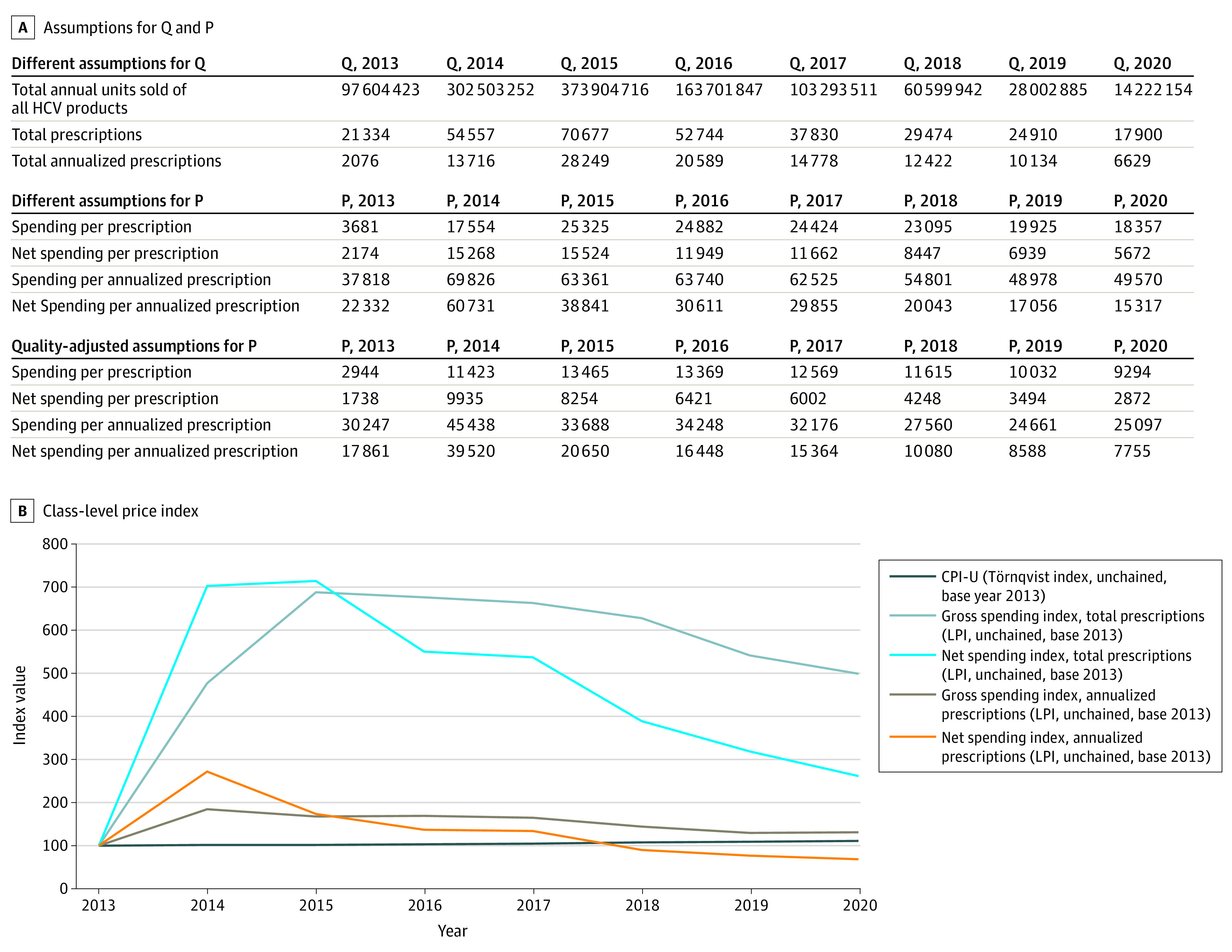
Class-Level Price Index for Hepatitis C Medications, With and Without Duration Adjustments, 2013 to 2020 CPI-U denotes Consumer Price Index for All Urban Consumers; HCV, hepatitis C virus; LPI, Laspeyres Price Index; P, price; and Q, quantity.

When we applied gross-to-net discount rates for HCV products using SSR Health data for the same period, the inflation rates were lower in both unadjusted and adjusted prescription models because rebate levels increased (Table 3). Before annualized prescription adjustments, the class-level net price index showed that prices increased 160% from 2013 to 2020 (Figure 2); however, when we included our duration adjustment, the price index showed that prices fell by 31% (Table 3 and Figure 2). In this case, the price was lower when rebates and duration were considered.

## Discussion

This article evaluates 3 concerns regarding existing price indexes for pharmaceuticals: (1) accounting for new drugs entering the market, (2) adjusting for manufacturer rebates and discounts to compare gross price changes with net price changes, and (3) adjusting product quantity definitions to account for treatment duration. With the increased focus on drug price inflation after the passage of the IRA, it is imperative that more sophisticated methods of measuring increases in drug prices be developed.

In this case study, a product-level approach underestimated the rate of inflation by failing to account for a new product that captured more than 14% of drug spending for HCV in its first year. Using gross prices for drugs (ie, wholesale acquisition cost or list price) may be inappropriate or misleading considering the substantial reductions obtained through rebate and discount negotiations by payers for brand products and by wholesalers and group purchasing organizations for generic products.

For any drug price changes in Medicare Parts B and D, ASP and AMP should be used by the Centers for Medicare & Medicaid Services. When gross prices are used, net spending amounts should be assessed simultaneously to better understand inflation in the context of manufacturer rebates and discounts. In the case of HCV treatment, the rate of net spending was considerably lower than gross spending, probably representing net price declines. Lastly, the study analyses demonstrated that a simple “per prescription” definition would substantially overestimate the rate of inflation for HCV drugs by failing to adjust for the shorter duration required (only 2-3 months) to reach a sustained virologic response vs the previous 12-month treatment requirement. The definition of a prescription may vary because a practitioner may prescribe a 12-month regimen as a single prescription; however, it is typically operationalized in claims databases by payer limits on days’ supplies. In our analysis, annualizing the prescription definition better reflected the course of treatment a patient would receive from the different products within the data set.

Currently, the IRA requires that prices for individual drug products not outpace the growth of the Consumer Price Index.^1^ This is a considerable change given that many drugs are increasing their list prices at several times the overall inflation rate each year.^22^ Although this policy limits substantial price increases for existing drugs, it fails to account for a new product in a specific disease class that may drastically increase the average cost of treatment. In this HCV case study, we observed price inflation soon after the launch of novel products in 2014; however, we subsequently observed deflation across multiple calculation methods from 2016 to 2020 as these new expensive drugs lowered their prices. Policy makers should consider alternatives to price benchmarking, potentially focusing each analysis on a single class or disease area. Otherwise, the public will experience price increases, but the indexes will show the opposite.

Innovation in the pharmaceutical market produces new products and the discontinuation of previous products. Federal regulations move some drugs from more expensive branded status to much less expensive generic formulations. When a new therapy offers a clinical cure over a shorter regimen duration, as was the case with HCV therapy, the change in clinical practice must be considered. The study analysis shows that adjustments to capture prescription duration can substantially affect price index estimates. Different data sources to measure quantity can also affect inflation indexes as can different price measures. The goal should be to create an index that reflects the prices that people pay to treat that condition.

### Limitations

The benefits of new drugs were measured as a reduction in treatment duration, which is an inadequate measure of clinical efficacy. The new HCV drugs are associated with an important clinical improvement over previous drugs.^23^ Several quality advancements were observed during the past decade for patients with HCV, including increases in the sustained virologic response (ie, clinical cure) rate, fewer adverse effects, and lower pill burdens. Dunn and colleagues^24^ and Cutler and colleagues^25^ have both proposed utility-based quality adjustments for assessing drug price inflation that could be combined with our class-based, prescription-adjusted approach. However, this is a separate topic requiring a separate analysis.

Another limitation was the sample selection using 2013 to 2020 Medicare data for these products, which failed to capture spending before multiple direct-acting antiviral launches, including boceprevir, telaprevir, and sofosbuvir. Sofosbuvir launched on December 6, 2013; therefore, our 20% sample data included $2.8 million in Medicare Part D spending in 2013, with just 94 prescriptions. By including pre-2013 data, we may have observed more substantial overall price changes that accounted for these product launches.

The focus on a single therapeutic area, such as HCV, limits the generalizability to other conditions because HCV treatment is relatively straightforward, and these products are not typically used for multiple conditions. When applying this approach to products approved for different diseases (eg, pembrolizumab indicated for multiple cancers, semaglutide indicated for diabetes and for long-term weight management), researchers may need to use data containing patient-level diagnosis codes to map spending appropriately. The classification level (ie, therapeutic use, pharmacologic activity, anatomic system) used for the price index will affect the selection of products and generalizability of findings.

This study did not assess patient out-of-pocket costs. To assess drug price inflation for patients, out-of-pocket expenses may be useful for understanding how patients are affected at the pharmacy counter. Net price was estimated using an aggregated gross-to-net discount rate for the entire class of HCV products from SSR Health. Although SSR Health data’s limitations have been described,^26^ these data have been shown to provide a reasonable estimation of market rebates for the purposes of our index demonstration.^27,28^

## Conclusions

This cross-sectional study found that existing product-level methods to estimate drug price inflation are likely underestimating price increases within a given disease area by failing to account for high launch prices of new market entrants. Using a class-level approach, the index can better capture any higher spending on new products at launch. Other therapeutic areas should be reviewed and assessed for necessary adjustments needed for accurate price comparisons. The ultimate goal is to have an inflation index that includes all drugs and incorporates improvements in outcomes. This would help to provide the most accurate answer to the question: “Are drug prices rising?”

## Notes

Supplement 1. **eTable 1.** Product-specific clinical information across all medications in sample for hepatitis C from 2013-2020. **eTable 2.** Summary of all hepatitis C virus medications available in Medicare Part D claims from 2013 to 2020. **eTable 3.** Total prescriptions (Rx) adjusted by lowest regimen duration for one year.
eReferences.

Supplement 2.
Data Sharing Statement
