# Supplementary material for: Comparison of Price Index Methods and Drug Price Inflation Estimates for Hepatitis C Virus Medications
Source: JAMA Health Forum. 2023 Jun 9;4(6):e231317. doi: 10.1001/jamahealthforum.2023.1317 (PMC10257099; doi:10.1001/jamahealthforum.2023.1317)
Supplement: Supplement 1. — eTable 1. Product-specific clinical information across all medications in sample for hepatitis C from 2013-2020. eTable 2. Summary of all hepatitis C virus medications available in Medicare Part D claims from 2013 to 2020. eTable 3. Total prescriptions (Rx) adjusted by lowest regimen duration for one year. eReferences. [file jamahealthforum-e231317-s001.pdf]

Supplemental Online Content

Mattingly II TJ, Anderson GF, Levy JF. Comparison of price index methods and drug price inflation estimates for hepatitis C virus medications. *JAMA Health Forum.* 2023;4(6):e231317. doi:10.1001/jamahealthforum.2023.1317

eTable 1. Product-specific clinical information across all medications in sample for Hepatitis C from 2013-2020.

eTable 2. Summary of all hepatitis C virus medications available in Medicare Part D claims from 2013 to 2020.

eTable 3. Total prescriptions (Rx) adjusted by lowest regimen duration for one year.

eReferences.

This supplemental material has been provided by the authors to give readers additional information about their work.

© 2023 Mattingly II TJ. *JAMA Health Forum.*

eTable 1. Product-specific clinical information across all medications in sample for Hepatitis C from 2013-2020.

Product Name	Active Ingredient	Typical Dosing	Need Additional Product	Minimum Duration (Days)	Duration (Months)	Sources
Copegus	ribavirin	800 mg daily ORALLY (in 2 divided doses) plus peginterferon alfa-2a 180 mcg SUBQ once weekly for 48 weeks	Yes	336	12	[1,2]
Daklinza	daclatasvir	60 mg orally once daily in combination with sofosbuvir for 12 weeks	Yes	84	3	[1,3]
Epclusa	Sofosbuvir / velpatasvir	Sofosbuvir 400 mg/velpatasvir 100 mg (one 400 mg/100 mg tablet) orally once daily for 12 weeks	No	84	3	[1,3]
Harvoni	Ledipasvir / sofosbuvir	Ledipasvir 90 mg/sofosbuvir 400 mg orally once daily for 12 weeks; may consider 8 weeks of treatment in patients without cirrhosis if pretreatment hepatitis C virus (HCV) RNA level is less than 6 million international units/mL	No	56	2	[1,3]
Incivek	telaprevir	750 mg taken 3 times a day; must be administered with both peginterferon alfa and ribavirin for all patients for 12 weeks, followed by a response-guided regimen of either 12 or 36 additional weeks of peginterferon alfa and ribavirin depending on viral response and prior response status	Yes	168	6	[4,5]
Ledipasvir / sofosbuvir	Ledipasvir / sofosbuvir	Ledipasvir 90 mg/sofosbuvir 400 mg orally once daily for 12 weeks; may consider 8 weeks of treatment in patients without cirrhosis if pretreatment hepatitis C virus (HCV) RNA level is less than 6 million international units/mL	No	56	2	[1,3]
Mavyret	Glecaprevir / pibrentasvir	Glecaprevir 300 mg/pibrentasvir 120 mg (three 100 mg/40 mg tablets) orally once daily with food for 8 weeks	No	56	2	[1,3]
Moderiba	ribavirin	800 mg daily ORALLY (in 2 divided doses) plus peginterferon alfa-2a 180 mcg SUBQ once weekly for 48 weeks	Yes	336	12	[1,2]
Moderiba 1200 Dose Pack	ribavirin	800 mg daily ORALLY (in 2 divided doses) plus peginterferon alfa-2a 180 mcg SUBQ once weekly for 48 weeks	Yes	336	12	[1,2]

© 2023 Mattingly TJ. *JAMA Health Forum.*

Moderiba 800 Dose Pack	ribavirin	800 mg daily ORALLY (in 2 divided doses) plus peginterferon alfa-2a 180 mcg SUBQ once weekly for 48 weeks	Yes	336	12	[1,2]
Olysio	simeprevir	150 mg orally once daily for 12 weeks in combination with sofosbuvir	Yes	84	3	[1,6]
Pegasys	peginterferon alfa-2a	180 mcg subQ once a week; duration of therapy is 48 weeks in combination with ribavirin only	Yes	336	12	[1,2]
Pegasys Proclick	peginterferon alfa-2a	180 mcg subQ once a week; duration of therapy is 48 weeks in combination with ribavirin only	Yes	336	12	[1,2]
Pegintron	peginterferon alfa-2b	180 mcg subQ once a week; duration of therapy is 48 weeks in combination with ribavirin only	Yes	336	12	[1,2]
Rebetol	ribavirin	800 mg daily ORALLY (in 2 divided doses) plus peginterferon alfa-2a 180 mcg SUBQ once weekly for 48 weeks	Yes	336	12	[1,2]
Ribasphere	ribavirin	800 mg daily ORALLY (in 2 divided doses) plus peginterferon alfa-2a 180 mcg SUBQ once weekly for 48 weeks	Yes	336	12	[1,2]
Ribasphere Ribapak	ribavirin	800 mg daily ORALLY (in 2 divided doses) plus peginterferon alfa-2a 180 mcg SUBQ once weekly for 48 weeks	Yes	336	12	[1,2]
Ribavirin	ribavirin	800 mg daily ORALLY (in 2 divided doses) plus peginterferon alfa-2a 180 mcg SUBQ once weekly for 48 weeks	Yes	336	12	[1,2]
Sofosbuvir / velpatasvir	Sofosbuvir / velpatasvir	Sofosbuvir 400 mg/velpatasvir 100 mg (one 400 mg/100 mg tablet) orally once daily for 12 weeks	No	84	3	[1,3]
Sovaldi	sofosbuvir	400mg daily for 12 weeks	Yes	84	3	[1,6]
Sylatron	peginterferon alfa-2b	Monotherapy (In renal disease), 1 mcg/kg/wk subQ for 1 year	No	336	12	[1,2]
Technivie	Ombitasvir / paritaprevir / ritonavir	2 tablets (ombitasvir 12.5 mg/paritaprevir 75 mg/ritonavir 50 mg) orally once daily with a meal plus weight-based ribavirin	Yes	84	3	[1,6]
Victrelis	boceprevir	800 mg administered orally three times daily (every 7 - 9 hours) with food	Yes	168	6	[4,7]
Viekira Pak	dasabuvir; ombitasvir/	fixed-dose combination of paritaprevir (150 mg)/ritonavir (100 mg)/ombitasvir (25 mg) plus twice-daily dosed dasabuvir (250 mg) for 12 weeks.	No	84	3	[1,6]

© 2023 Mattingly TJ. *JAMA Health Forum.*

	paritaprevir/ ritonavir					
Viekira XR	Dasabuvir / ombitasvir/ paritaprevir/ ritonavir	fixed-dose combination of paritaprevir (150 mg)/ritonavir (100 mg)/ombitasvir (25 mg) plus twice-daily dosed dasabuvir (250 mg) for 12 weeks.	No	84	3	[1,6]
Vosevi	sofosbuvir/ velpatasvir/ voxilaprevir	Sofosbuvir 400 mg/velpatasvir 100 mg/voxilaprevir 100 mg orally once daily with food for 12 weeks	No	84	3	[1,3]
Zepatier	elbasvir/grazoprevir	Elbasvir 50 mg/grazoprevir 100 mg orally once daily for 12 weeks	No	84	3	[1,3]

Abbreviations: SUBQ-subcutaneously;

© 2023 Mattingly TJ. *JAMA Health Forum.*

eTable 2: Summary of all hepatitis C virus medications available in Medicare Part D claims from 2013 to 2020.

Active Ingredient (Product name or listed as "generic")	Number of Prescriptions	Average Day Supply (STD)	Average Quantity Dispensed (STD)	Average Med D Paid, US dollars (STD)	Average OOP, US dollars (STD)	Last Package AWP, US dollars	Last Package WAC, US dollars	Any Price Change	Last Price Change Date
Ribavirin (Copegus)	30	22.4 (9.8)	106.9 (75.9)	1,729 (1,223)	88 (129)	4,280.54	3,567.12	Y	1/1/16
Daclatasvir (Daklinza)	6,455	28.0 (0.1)	28.7 (2.1)	19,861 (1,356)	323 (389)	25,200.00	21,000.00	N	5/16/16
Sofosbuvir/Velpatasvir (Epclusa)	33,533	27.9 (0.2)	27.9 (0.2)	21,275 (308)	550 (47)	29,904.00	24,920.00	N	6/28/16
Ledipasvir/Sofosbuvir (Harvoni)	112,287	28.0 (0.2)	31.5 (9.9)	31,200 (10,533)	1,264 (1,675)	37,800.00	31,500.00	N	10/10/14
Telaprevir (Incivek)	1,794	28.3 (0.3)	166.8 (2.1)	19,012 (1,296)	256 (221)	26,462.04	22,051.70	Y	8/1/13
Ledipasvir/Sofosbuvir (generic)	730	28.5 (0.8)	28.5 (0.8)	9,215 (520)	503 (132)	14,400.00	12,000.00	N	12/17/18
Glecaprevir/pibrentasvir (Mavyret)	20,539	28.1 (0.1)	84.2 (0.2)	10,583 (355)	297 (172)	15,840.00	13,200.00	N	8/3/17
Ribavirin (Moderiba)	199	28.0 (0.2)	134.5 (10.4)	171 (8)	3 (1)	474.00	395.00	N	1/20/14
Ribavirin (Moderiba 1200 Dose Pack)	232	22.8 (10.5)	48.4 (13.6)	775 (308)	45 (65)	1,384.02	1,153.35	N	1/20/14
Ribavirin (Moderiba 800 Dose Pack)	54	26.6 (2.7)	53.2 (4.9)	597 (96)	48 (25)	922.68	768.90	N	1/20/14
Simeprevir (Olysio)	8,742	28.1 (0.2)	28.1 (0.2)	20,111 (411)	354 (261)	26,544.00	22,120.00	N	11/26/13
Peginterferon alfa-2a (Pegasys)	10,019	30.5 (3.2)	2.8 (1.1)	2,693 (479)	214 (93)	4,903.15	4,085.96	Y	1/1/18
Peginterferon alfa-2a (Pegasys Proclick)	6,275	36.3 (16.0)	2.6 (1.1)	2,833 (696)	79 (51)	4,903.15	4,085.96	Y	1/1/18
Peginterferon alfa-2b (Pegintron)	3,061	30.1 (12.7)	5.0 (5.0)	2,654 (1,602)	157 (310)	4,179.30	3,482.75	Y	8/1/14
Ribavirin (Rebetol)	19	21.8 (8.2)	378.3 (296.7)	635 (556)	14 (27)	232.80	194.00	N	1/14/04
Ribavirin (Ribasphere)	11,202	27.4	114.0 (41.7)	263 (284)	11 (12)	474.00	395.00	Y	10/6/11
Ribavirin (Ribaspher Ribapak)	5,846	28.1 (0.2)	56.2 (0.3)	1,059 (376)	36 (39)	2,698.85	2,249.04	Y	7/1/15
Ribavirin (generic)	27,591	26.3 (4.5)	122.8 (32.8)	145 (64)	4 (6)	1,390.00	383.00	N	12/5/05
Sofosbuvir/velpatasvir (generic)	3,867	28.5 (0.3)	28.6 (0.3)	6,029 (194)	337 (62)	9,600.00	8,000.00	N	12/17/18
Sofosbuvir (Sovaldi)	40,287	28.2 (0.7)	28.2 (0.7)	25,802 (812)	424 (229)	33,600.00	28,000.00	N	12/6/13
Peginterferon alfa-2b (Sylatron)	366	29.8 (7.9)	3.6 (1.5)	7,071 (5,052)	460 (852)	7,128.86	5,940.72	Y	1/5/18
Ombitasvir/paritaprevir/ritonavir (Technivie)	36	28.0 (0.0)	56.0 (0.0)	23,016 (741)	399 (375)	30,661.20	25,551.00	N	8/1/15
Boceprevir (Victrelis)	1,718	27.9 (0.3)	334.4 (3.5)	4,643 (571)	81 (70)	8,024.06	6,686.72	Y	12/31/13
Dasabuvir/ombitasvir/paritaprevir/ritonavir (Viekira Pak)	2,288	28.2 (0.4)	111.7 (0.7)	23,227 (1,194)	788 (821)	33,327.60	27,773.00	N	12/19/14
Dasabuvir/ombitasvir/paritaprevir/ritonavir (Viekira XR)	230	29.6 (2.7)	84.0 (0.0)	23,943 (1,805)	361 (326)	33,327.60	27,773.00	N	7/26/16
Sofosbuvir/velpatasvir/voxilaprevir (Vosevi)	3,099	28.0 (0.2)	28.0 (0.2)	21,807 (453)	547 (44)	29,904.00	24,920.00	N	7/18/17
Elbasvir/grazoprevir (Zepatier)	8,927	24.9 (4.2)	24.9 (4.2)	10,515 (4,565)	253 (166)	8,736.00	7,280.00	Y	10/4/18

Abbreviations: STD-standard deviation; OOP-out-of-pocket; AWP-average wholesale price; WAC-wholesale acquisition cost

© 2023 Mattingly TJ. *JAMA Health Forum.*

eTable 3. Total prescriptions (Rx) adjusted by lowest regimen duration for one year.

Active Ingredient (Product name or listed as "generic")	Rx Per Patient	2013	Adj Rx	2014	Adj Rx	2015	Adj Rx	2016	Adj Rx	2017	Adj Rx	2018	Adj Rx	2019	Adj Rx	2020	Adj Rx
Ribavirin (Copegus)	12	26	2.17	3	0.25	1	0.08	NA	NA	NA	NA	NA	NA	NA	NA	NA	NA
Daclatasvir (Daklinza)	3	NA	NA	NA	NA	1,211	404	3,898	1,299	1,289	430	51	17	6	2	NA	NA
Sofosbuvir/Velpatasvir (Epclusa)	3	NA	NA	NA	NA	NA	NA	3,103	1,034	7,554	2,518	7,549	2,516	8,194	2,731	7,133	2,378
Ledipasvir/Sofosbuvir (Harvoni)	2	NA	NA	4,408	2,204	45,455	22,728	28,681	14,341	16,627	8,314	11,036	5,518	4,559	2,280	1,521	761
Telaprevir (Incivek)	6	1,722	287	72	12	NA	NA	NA	NA	NA	NA	NA	NA	NA	NA	NA	NA
Ledipasvir/Sofosbuvir (generic)	2	NA	NA	NA	NA	NA	NA	NA	NA	NA	NA	NA	NA	400	200	330	165
Glecaprevir/pibrentasvir (Mavyret)	2	NA	NA	NA	NA	NA	NA	NA	NA	1,094	547	7,002	3,501	8,151	4,076	4,292	2,146
Ribavirin (Moderiba)	12	NA	NA	125	10.42	48	4	26	2	NA	NA	NA	NA	NA	NA	NA	NA
Ribavirin (Moderiba 1200 Dose Pack)	12	NA	NA	134	11	75	6	22	2	NA	NA	1	0.08	NA	NA	NA	NA
Ribavirin (Moderiba 800 Dose Pack)	12	NA	NA	26	2	23	2	5	0.42	NA	NA	NA	NA	NA	NA	NA	NA
Simeprevir (Olysio)	3	12	4	7,397	2,466	1,042	347	223	74	66	22	2	0.67	NA	NA	NA	NA
Peginterferon alfa-2a (Pegasys)	12	4,463	372	2,131	178	381	32	375	31	475	40	549	46	713	59	932	78
Peginterferon alfa-2a (Pegasys Proclick)	12	3,807	317	2,305	192	64	5	8	0.67	27	2	39	3	25	2	NA	NA
Peginterferon alfa-2b (Pegintron)	12	1,479	123	1,425	119	127	11	14	1	10	1	4	0.33	2	0.2	NA	NA
Ribavirin (Rebetol)	12	2	0.2	2	0.2	9	1	1	0.08	1	0.08	2	0.2	2	0.2	NA	NA
Ribavirin (Ribasphere)	12	2,563	214	3,264	272	2,908	242	1,778	148	511	43	173	14	5	0.4	NA	NA
Ribavirin (Ribaspher Ribapak)	12	1,508	126	3,154	263	877	73	262	22	41	3.4	4	0.3	NA	NA	NA	NA
Ribavirin (generic)	12	4,029	336	8,016	668	6,954	580	4,530	378	2,048	171	840	70	661	55	513	43
Sofosbuvir/velpatasvir (generic)	3	NA	NA	NA	NA	NA	NA	NA	NA	NA	NA	NA	NA	1,263	421	2,604	868
Sofosbuvir (Sovaldi)	3	94	31	21,851	7,284	9,766	3,255	6,926	2,309	1,524	508	70	23	23	8	33	11
Peginterferon alfa-2b (Sylatron)	12	86	7	71	6	73	6	68	6	31	3	26	2	10	1	1	0.08
Ombitasvir/paritaprevir/ritonavir (Technivie)	3	NA	NA	NA	NA	25	8	9	3	2	0.7	NA	NA	NA	NA	NA	NA
Boceprevir (Victrelis)	6	1,543	257	172	29	3	0.5	NA	NA	NA	NA	NA	NA	NA	NA	NA	NA
Dasabuvir/ombitasvir/paritaprevir/ritonavir (Viekira Pak)	3	NA	NA	1	0.3	1,635	545	579	193	70	23	3	1	NA	NA	NA	NA
Dasabuvir/ombitasvir/paritaprevir/ritonavir (Viekira XR)	3	NA	NA	NA	NA	NA	NA	75	25	149	50	6	2	NA	NA	NA	NA
Sofosbuvir/velpatasvir/voxilaprevir (Vosevi)	3	NA	NA	NA	NA	NA	NA	NA	NA	660	220	1202	400	729	243	508	169
Elbasvir/grazoprevir (Zepatier)	3	NA	NA	NA	NA	NA	NA	2,161	720.33	5,651	1,884	915	305	167	56	33	11
**Grand Total**		**21,334**	**2,076**	**54,557**	**13,716**	**70,677**	**28,249**	**52,744**	**20,589**	**37,830**	**14,778**	**29,474**	**12,422**	**24,910**	**10,134**	**17,900**	**6,629**

© 2023 Mattingly TJ. *JAMA Health Forum.*

Appendix References

1. IBM. IBM Micromedex. https://www.micromedexsolutions.com. Published 2022. Accessed September 1, 2022.
2. Ghany MG, Strader DB, Thomas DL, Seeff LB. Diagnosis, management, and treatment of hepatitis C: An update. *Hepatology*. 2009;49(4):1335-1374. doi:10.1002/hep.22759
3. AASLD-IDSA HCV Guidance Panel. Hepatitis C Guidance 2018 Update: AASLD-IDSA Recommendations for Testing, Managing, and Treating Hepatitis C Virus Infection. *Clin Infect Dis*. 2018;67(10):1477-1492. doi:10.1093/cid/ciy585
4. Butt AA, Kanwal F. Boceprevir and Telaprevir in the Management of Hepatitis C Virus–Infected Patients. *Clin Infect Dis*. 2012;54(1):96-104. doi:10.1093/cid/cir774
5. Vertex Pharmaceuticals Inc. *INCIVEK [Package Insert]*. Cambridge, MA; 2011.
6. Chung RT, Davis GL, Jensen DM, et al. Hepatitis C guidance: AASLD-IDSA recommendations for testing, managing, and treating adults infected with hepatitis C virus. *Hepatology*. 2015;62(3):932-954. doi:10.1002/hep.27950
7. Merck & Co. Inc. *VICTRELIS [Package Insert]*. Whitehouse Station, NJ; 2011.

© 2023 Mattingly TJ. *JAMA Health Forum.*
