# Supplementary material for: Comparison of Price Index Methods and Drug Price Inflation Estimates for Hepatitis C Virus Medications
Source: JAMA Health Forum. 2023 Jun 9;4(6):e231317. doi: 10.1001/jamahealthforum.2023.1317 (PMC10257099; doi:10.1001/jamahealthforum.2023.1317)
Supplement: Supplement 2. — Data Sharing Statement [file jamahealthforum-e231317-s002.pdf]

Data Sharing Statement

Mattingly, II. Comparison of Price Index Methods and Drug Price Inflation Estimates for Hepatitis C Virus Medications. *JAMA Health Forum*. Published June 09, 2023. doi:10.1001/jamahealthforum.2023.1317

Data
Data available: No

Additional Information
Explanation for why data not available: Individual Medicare beneficiary data is maintained by RESDAC. All data has been aggregated and provided as supplementary material.
